# Microstructure and Solidification Crack Susceptibility of Al 6014 Molten Alloy Subjected to a Spatially Oscillated Laser Beam

**DOI:** 10.3390/ma11040648

**Published:** 2018-04-23

**Authors:** Minjung Kang, Heung Nam Han, Cheolhee Kim

**Affiliations:** 1 Welding and Joining Research Group, Korea Institute of Industrial Technology, 7-47, Songdodong, Yeonsugu, Incheon 21999, Korea; kmj1415@kitech.re.kr; 2 Department of Materials Science and Engineering and RIAM, Seoul National University, Seoul 08826, Korea; hnhan@snu.ac.kr

**Keywords:** laser welding, single mode laser, beam oscillation, Al alloy, solidification cracking susceptibility, microstructure, solidification morphology

## Abstract

Oscillating laser beam welding for Al 6014 alloy was performed using a single mode fiber laser and two-axis scanner system. Its effect on the microstructural evolution of the fusion zone was investigated. To evaluate the influence of oscillation parameters, self-restraint test specimens were fabricated with different beam patterns, widths, and frequencies. The behavior of hot cracking propagation was analyzed by high-speed camera and electron backscatter diffraction. The behavior of crack propagation was observed to be highly correlated with the microstructural evolution of the fusion zone. For most oscillation conditions, the microstructure resembled that of linear welds. A columnar structure was formed near the fusion line and an equiaxed structure was generated at its center. The wide equiaxed zone of oscillation welding increased solidification crack susceptibility. For an oscillation with an infinite-shaped scanning pattern at 100 Hz and 3.5 m/min welding speed, the bead width, solidification microstructure, and the width of the equiaxed zone at the center of fusion fluctuated. Furthermore, the equiaxed and columnar regions alternated periodically, which could reduce solidification cracking susceptibility.

## 1. Introduction

Solidification cracking is a well-known defect frequently observed in aluminum welds. The low ductility of a semi-solid in the mushy zone and the high solidification shrinkage of aluminum alloys both increase hot cracking susceptibility [1,2]. Solidification cracking is initiated by complex interactions between metallurgical and mechanical factors [3]. During laser welding, this can be diminished by improving chemical composition, refining solidification structure, optimizing laser pulsing parameters, and/or reducing thermal strains [4]. The chemical composition of welds can be easily improved by adding a filler wire, a method utilized in arc welding processes to reduce solidification cracking. However, this is not always practical, particularly in laser welding, because additional wire feeding can deteriorate the flexibility of the process.

Solidification cracking has been evaluated for various fusion welding processes such as arc welding [5,6,7], electron beam welding [8], and laser beam welding [9,10,11,12]. Laser welding using a high-density laser beam can reduce heat input to a workpiece and minimize thermal distortion. Recently, a high-speed laser beam oscillation technique using galvanometric scanners was developed, allowing the use of wide weld beads and controlling the thermal history of welds. Process flexibility is also guaranteed by a high moving speed with a small heat input. Its application to industrial materials such as steel [13,14], aluminum [15,16,17,18], and copper [19] has been attempted. The use of dynamic oscillation techniques leads to the stabilization of the welding process in terms of welding depth and molten pool behavior [15]. Laser beam oscillation regulates the shape of heat flow, temperature gradient, and solidification rate [20]. Wang et al. controlled the fraction of equiaxed microstructure in the fusion zone by applying laser beam oscillation [21]. A single mode laser with high brilliance is gradually being applied in welding. The high energy density of a single mode laser beam enhances the laser scan speed and oscillation frequency, which could have unique applications [22,23]. Previous studies have demonstrated how laser beam oscillation affected weld morphology. However, the influence of high speed beam oscillation on hot cracking susceptibility is still insufficiently discussed.

Kou and Le [24] suggested that modifying the microstructure by welding arc oscillation hindered solidification cracking. The direction of columnar dendritic grains changed periodically with the heat source oscillation, which made crack propagation difficult. Gollnow et al. [25] reported that the angle between the fusion line and the predominant main axis grains is correlated to the probability of occurrence of hot cracking. Shinozaki et al. [26] suggested that a coarse equiaxed grain structure resulted in high solidification cracking susceptibility, which was also confirmed by Tang and Vollertsen [27]. They added the AlTi5B1 alloy in the weld metal to refine its grains and observed that an optimum grain size minimized cracking susceptibility. Witzendorf et al. [28,29] evaluated the effects of relevant solidification parameters like temperature gradient and local grain growth rate during pulse laser welding. They insisted that the interface velocity (solidification rate) significantly influences hot cracking susceptibility since the temperature gradient at the final stage of solidification was hardly affected by other process parameters. Using a multimode laser with a beam diameter of 0.6 mm, Choi et al. [30] applied a low-frequency laser oscillation on the 6K21 Al alloy to improve its joint strength. The solidification crack was minimized at an oscillation frequency of 5 Hz. However, the molten pool overlapped at 20 Hz, and the joining strength degraded. Consequently, Kim et al. [31] developed a numerical analysis model to predict the morphology in low-frequency laser oscillation welding.

Since the microstructure formation influences hot cracking susceptibility, a good understanding of the relationship between grain structure and hot cracking is necessary. In this study, laser beam oscillation for the welding of Al 6014 alloy was performed with a single mode laser and a beam scanning system. Its effect on the microstructural evolution of the fusion zone and hot cracking susceptibility were investigated.

## 2. Experimental Procedure

**Figure 1:**
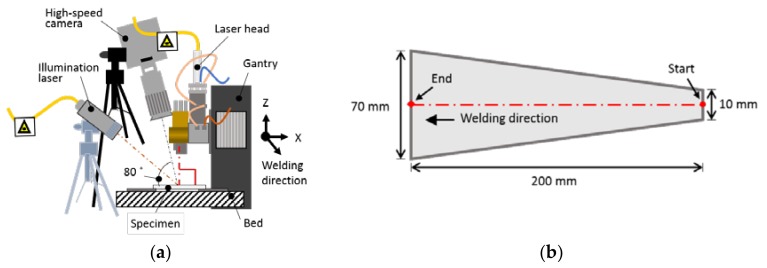
(**a**) Experimental setup for oscillating laser beam welding; (**b**) Schematic image of the self-restraint test specimen [8] to evaluate hot cracking susceptibility.

For the welding process, a single mode Yb:YAG laser, YLR-1000-SM-WC (IPG Photonics, Oxford, MA, USA), was used. The laser beam was delivered through an optical fiber with a diameter of 13 μm. The path was implemented by a single-axis translation system and two-axis beam scanner, D50 (IPG Photonics, Oxford, MA, USA), with a focal length of 250 mm (Figure 1a). The laser beam was perpendicularly irradiated to the specimen and was focused on the upper surface of the workpiece with a beam diameter of 41 μm. A shielding gas was not present during welding. To evaluate the hot cracking susceptibility under different welding conditions, a self-restraint test specimen was fabricated (Figure 1b) [8]. Laser welding initiated at the narrow edge and ended at the wide one. The dimensions of the specimen are shown in Figure 1b. The base material was a 1 mm-thick Al 6014-T4 alloy. The chemical composition of the base material is shown in Table 1. 

**Figure 2:**
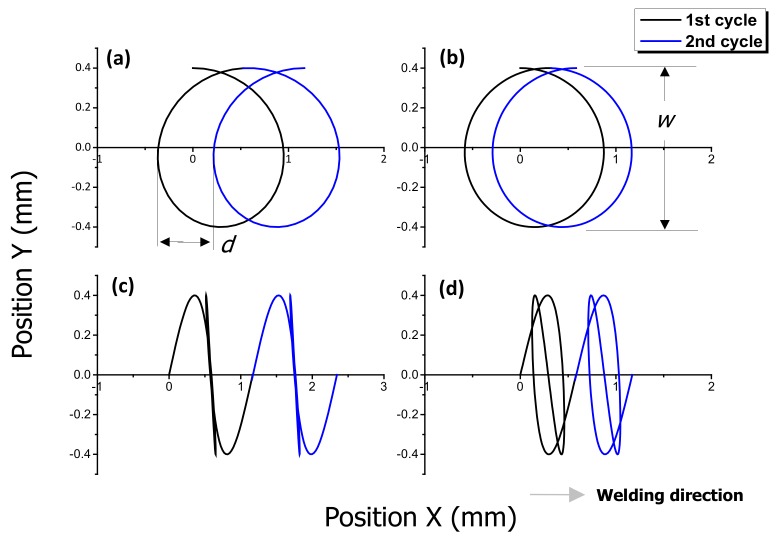
Circular and infinite-shaped laser oscillation beam patterns at a welding speed of 3.5 m/min, where *w* is the oscillation width at 0.8 mm and *d* is the interval between the periodic waves. (**a**) circular pattern at 100 Hz; (**b**) circular pattern at 200 Hz; (**c**) infinite-shaped pattern at 100 Hz; (d) infinite-shaped pattern at 200 Hz.

The beam scanner, equipped with two galvanometers to rotate the mirrors, can generate various oscillation patterns. In this study, circular and infinite-shaped patterns were adopted instead of general linear motion. To investigate the influence of laser beam oscillation, the oscillation width and frequency were varied. Figure 2 shows the laser beam path for the circular and infinite-shaped beam patterns at different frequencies. The single-axis translation system linearly moves the scanner along the x-axis, with a fixed travel speed (welding speed). The laser beam path is determined by considering the position of the scanner and the two-dimensional beam motion (circle or infinite-shaped) of the scanner. The details of the laser welding conditions are presented in Table 2. After welding, a non-destructive X-ray test was conducted to measure the hot cracking length using XSCAN-H160 (XAVIS, Seongnam, Gyeonggi, Korea). During welding, the molten pool surface was observed by a high-speed camera UX50 (Photron, San Diego, CA, USA) with a diode laser illumination LIMO120-F400 (Limo, Bookenburgweg, Dortmund, Germany) at a rate of 2000 frames per second. The high-speed camera was tilted by an angle of 80° relative to the specimen. An illumination 808 nm laser of power 100 W and a bandpass filter that transmits radiation in the range 808 ± 1.5 nm were used. The details of equipment applied in the experimental was summarized in Table 3.

After welding, horizontally sectioned specimens were prepared for microstructure observation. These were polished and etched with a solution of 1 mL HF, 1.5 mL HCl, 2.5 mL HNO_3_, and 95 mL H_2_O. The microstructure of the welds was observed through field-emission scanning electron microscopy (FESEM), using a Quanta 200 F (FEI company, Hillsboro, OR, USA), with electron backscatter diffraction (EBSD), using a Digiview4 (EDAX, Mahwah, NJ, USA). The EBSD specimens were mechanically polished, and then electro-polished at room temperature in a solution of 10% perchloric acid and ethanol at an operating voltage of 22 V. The critical misorientation angle was set at 15° for grain identification. The data were interpreted using an orientation imaging microscopy analysis software.

## 3. Results and Discussion

### 3.1. Effects of Circular Beam Pattern on Hot Crack Susceptibility

**Figure 3:**
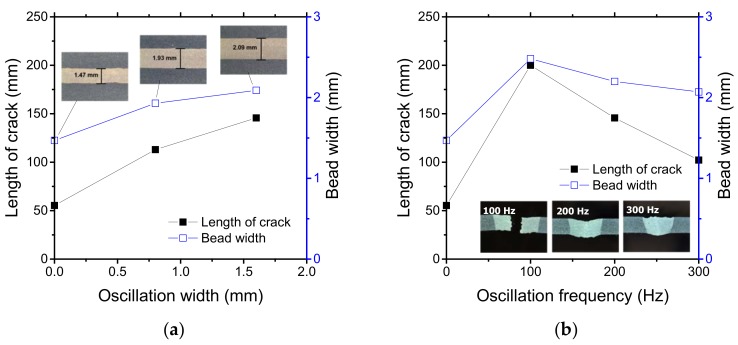
Crack length and bead width measurements at the surface according to (**a**) oscillation width and (**b**) frequency. Specimen was prepared using a circular scanning pattern under a laser power of 900 W and a welding speed of 3 m/min.

Oscillation width and frequency affected the hot cracking behavior. After welding with a circular beam pattern, longer cracks were observed at higher oscillation widths (Figure 3a). The width of the molten pool increased with the oscillation width. However, upon increasing the oscillation frequency, the hot cracking behavior varied. With an oscillation width of 1.6 mm, the longest crack was obtained at 100 Hz. Beyond this frequency, the length of the crack decreased correspondingly (Figure 3b). This confirmed the correlation of crack length with the bead width.

**Figure 4:**
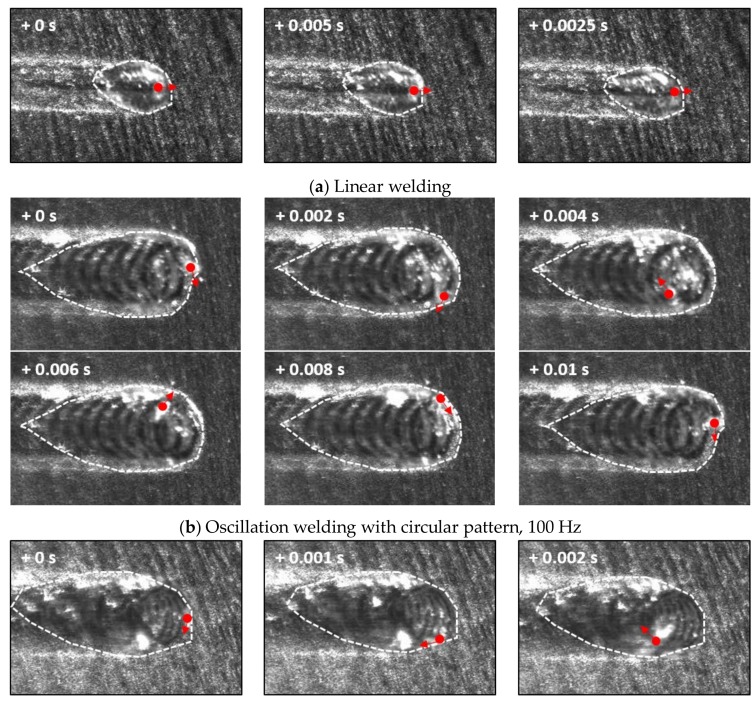

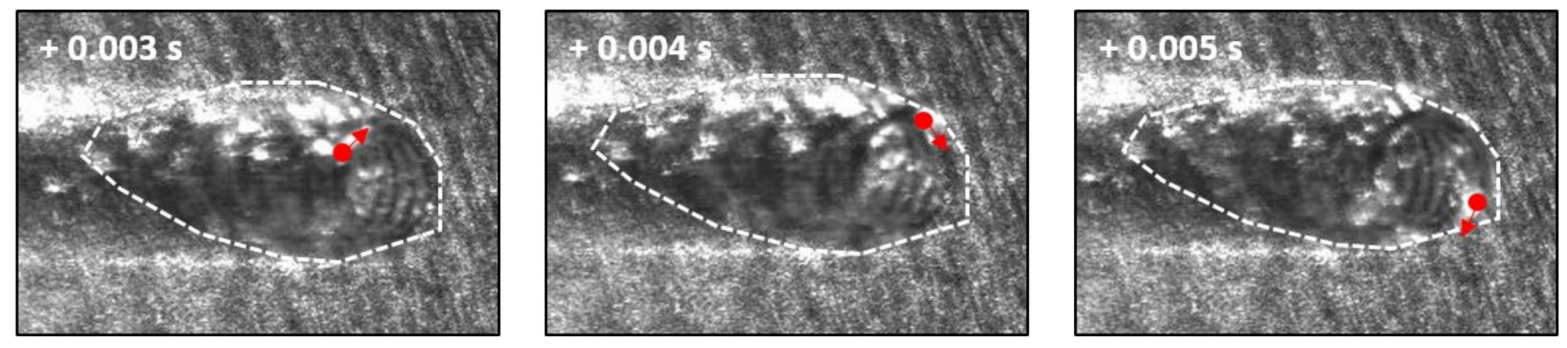
High-speed camera images of the molten pool with 900 W laser power at 3 m/min welding speed. The oscillation width was fixed at 1.6 mm, while the frequency varied at (**a**) 0 Hz (linear welding), (**b**) 100 Hz and (**c**) 200 Hz.

**Figure 5:**
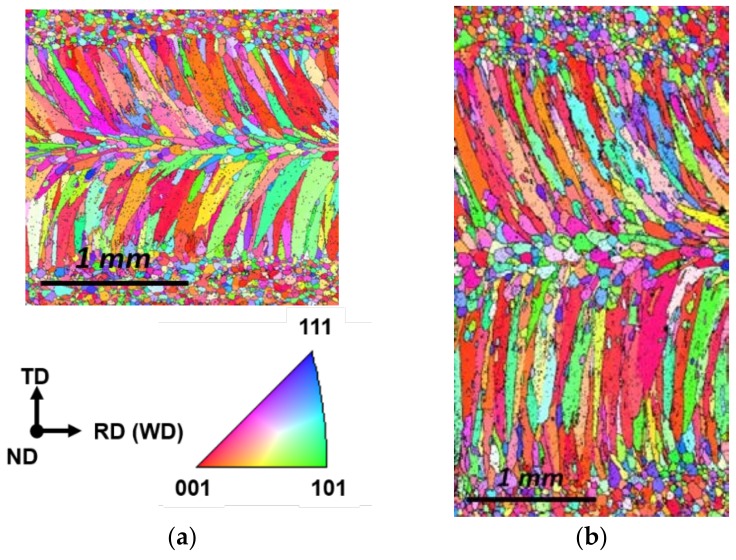
Electron backscatter diffraction (EBSD) analysis with and without laser beam oscillation. Specimen was prepared under laser power of 900 W at welding speed of 3 m/min. (**a**) linear welding; (**b**) oscillation welding with oscillation width of 1.6 mm at 200 Hz.

Molten pool size and shape directly influence hot cracking susceptibility [32]. The high-speed images of the molten pool for the circular beam scanning pattern can be seen in Figure 4. Both oscillation width and frequency affect the heat input to the specimen, which changes the molten pool behavior. The location of the keyhole and its moving direction are indicated by the red dot and arrow, respectively. Compared to linear welding (Figure 4a), a wider molten pool was produced by the oscillating laser beams (Figure 4b,c). A small keyhole generated by a single mode laser beam rapidly spins within the molten pool. However, the shape of the molten pool remained almost uniformly independent of the keyhole position. At the solidification boundary on the surface, the grain growth rates (R) and welding speed were equal at various oscillation frequencies, but the temperature gradient (G) was slightly different. Accordingly, the microstructural evolution within the welds due to the circularly oscillated beam was similar to that within the linear weld (Figure 5a). The columnar structure formed near the fusion line, while the equiaxed structure was generated at the center of the fusion zone. Furthermore, the resulting equiaxed zone was wider (Figure 5b) than that in the linear weld, because the temperature gradient is less in the circular patterns, which promotes the formation and growth of equiaxed structures [33]. The wide equiaxed zone along the centerline promoted the propagation of solidification cracks during welding [31]. Hence, the length of the crack increases with the oscillation width, as shown in Figure 3a. At high oscillation frequencies, the molten pool contracted (Figure 3b), which resulted in the reduction of solidification cracks.

### 3.2. Effects of Infinite-Shaped Beam Pattern on Hot Crack Susceptibility

**Figure 6:**
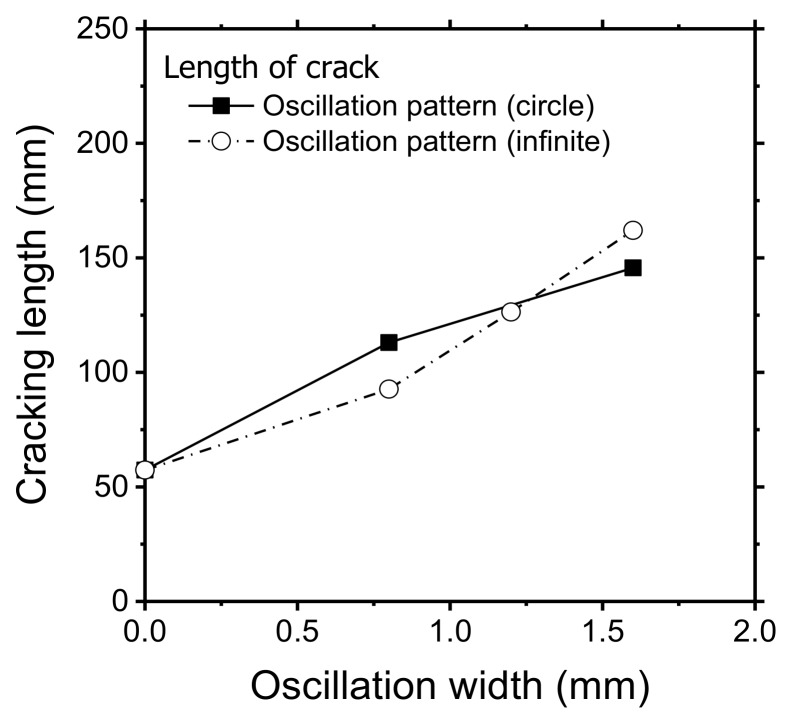
Measured length of the crack from different laser beam scanning patterns and oscillation widths (laser power: 900 W, oscillation frequency: 200 Hz, and welding speed: 3 m/min).

**Figure 7:**
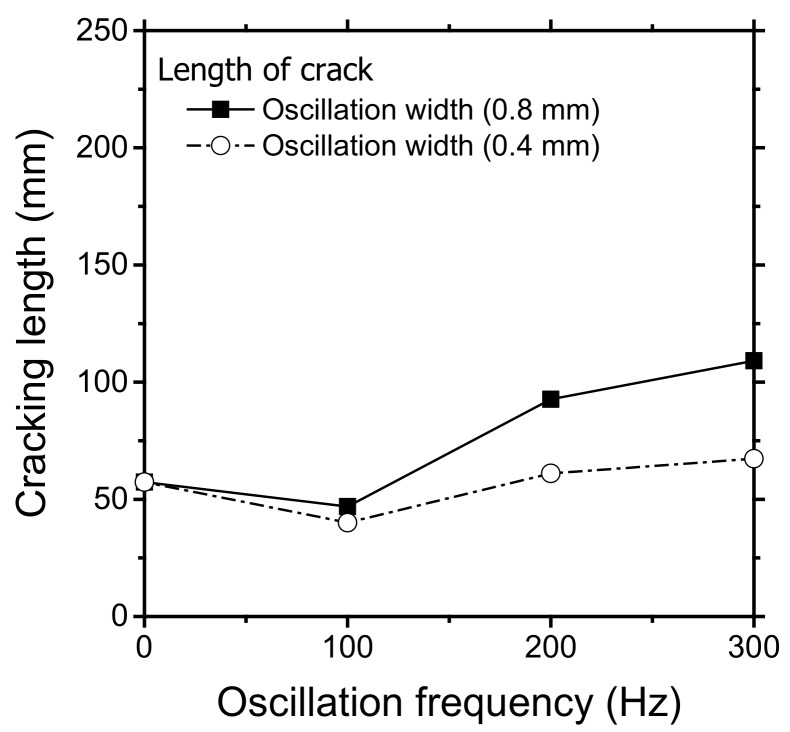
Measured length of the crack for different oscillation widths at varying frequencies. The specimen was prepared with an infinite-shaped pattern (laser power: 900 W, oscillation frequency: 200 Hz, and welding speed: 3 m/min).

**Figure 8:**
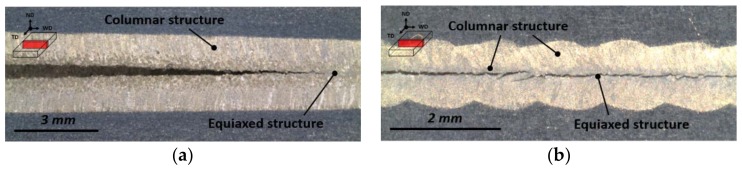
Bead appearance view from the normal direction depending on the welding condition (**a**). Circular scanning pattern with 900 W laser power and 1.6 mm oscillation width at 3 m/min welding speed and 100 Hz oscillation frequency and (**b**) infinite-shaped scanning pattern 900 W laser power and 0.4 mm oscillation width at 100 Hz and 3.5 m/min welding speed.

The cracking behavior for the infinite-shaped beam oscillation was similar to that for the circular beam oscillation. The crack length increased with the oscillation width at 200 Hz (Figure 6). In the experiments, the welding speed for most cases was 3.0 m/min, whereas the welding speed oscillations with width less than 0.8 mm was increased to 3.5 m/min to prevent overheating from heat accumulation. At varying oscillation frequencies, the shortest crack was observed at 100 Hz, exactly where the longest crack was observed for the circular pattern. Moreover, the crack length increased beyond 100 Hz. (Figure 7). The solidification crack propagated along the centerline for the self-restraint test with circular oscillation (Figure 8a), whereas the crack propagated in a zigzag manner with infinite-shaped oscillation (Figure 8b).

**Figure 9:**
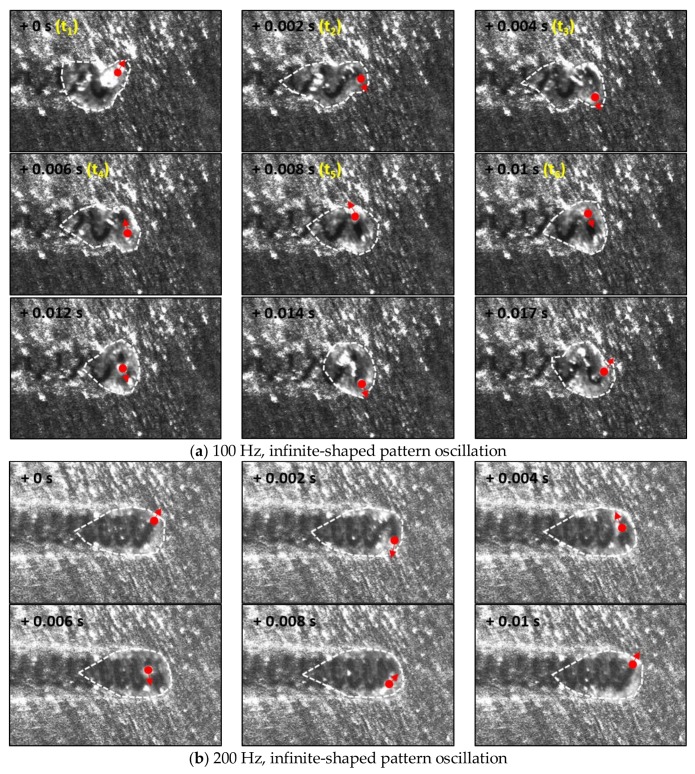
High-speed camera images of the molten pool under laser beam oscillation with an infinite-shaped pattern during a single oscillation. The oscillating laser beam welding was performed with 900 W laser power at 3.5 m/min. The oscillation width was fixed at 0.8 mm, while the frequency varied: (**a**) 100 and (**b**) 200 Hz.

**Figure 10:**
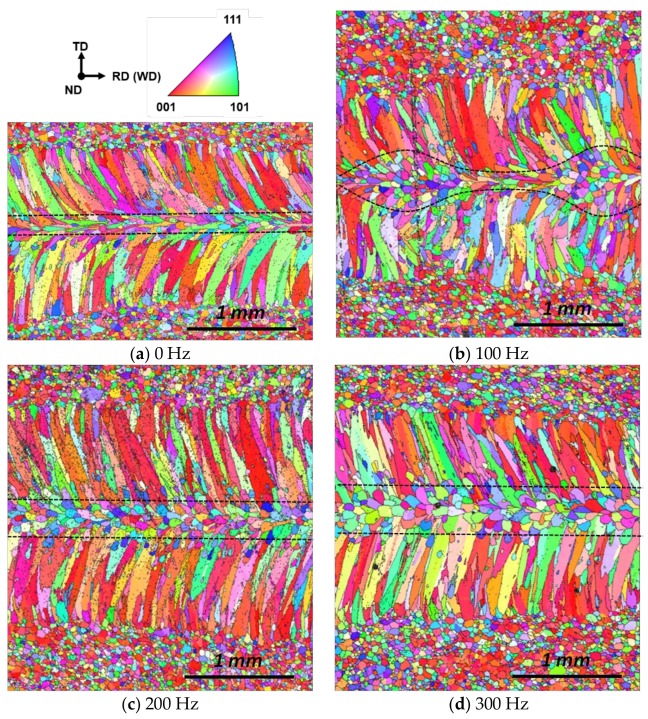
EBSD analysis with varying oscillation frequencies. Welding was performed with infinite-shaped beam pattern under 900 W laser power at 3.5 m/min welding speed. The oscillation width was fixed at 0.8 mm, while the frequency was varied at (**a**) 0 Hz; (**b**) 100 Hz; (**c**) 200 Hz and (**d**) 300 Hz.

The molten pool behavior for the infinite-shaped beam oscillation was also observed with a high-speed camera. At 100 Hz, the shape of the pool noticeably transforms depending on the position of the laser keyhole (Figure 9a). For nominal linear welding, the molten pool remained in a teardrop shape until the 100 Hz oscillation frequency. As shown in Figure 2c, the laser beam moves mostly transversely for some period, which was confirmed by the images taken between 0.006 s and 0.014 s, as shown in Figure 9a. As the oscillation frequency increased, the molten pool exhibited a stable teardrop shape regardless of the laser keyhole location (Figure 9b), similar to all the cases with the circular beam oscillation (Figure 4b,c). The grain growth direction is determined by the movement of the solid–liquid interface [33,34,35]. At 100 Hz oscillation frequency, the pool generated a repetitively fluctuating morphology (Figure 10b), as expected. The width of the equiaxed zone is inversely proportional to the bead width. With increasing oscillation frequency, the resulting fusion line straightened, resembling that in the linear weld. (Figure 10c,d). The equiaxed region at the center of fusion zone widened with the oscillation frequency as the temperature gradient was reduced.

**Figure 11:**
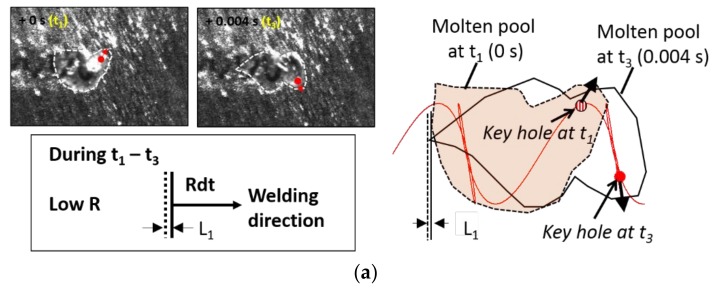

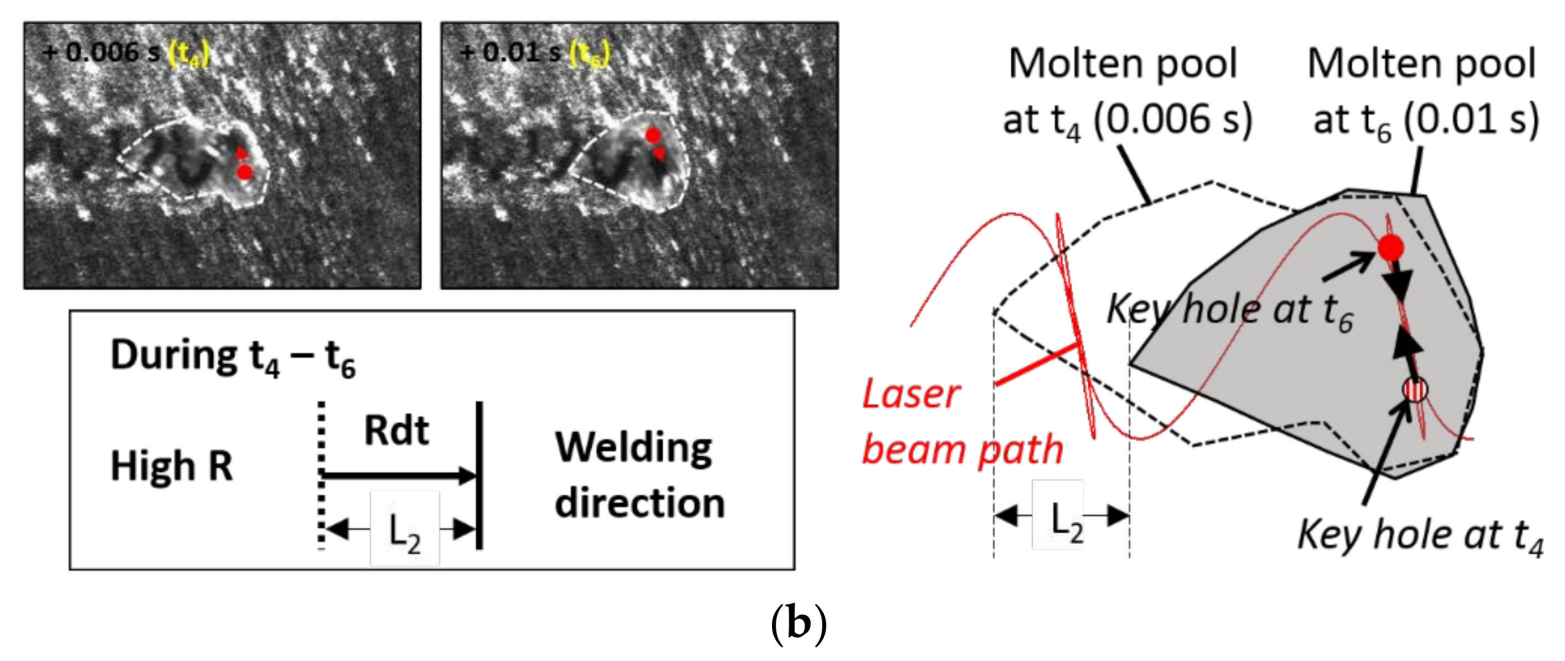
Schematic diagram of the molten pool movement during (**a**) t_1_–t_3_ and (**b**) t_4_–t_6_, where R represents the local solidification rate, W.D is the welding direction along the x-axis, and Rdt indicates the solid–liquid interface displacement over a time interval.

The oscillating morphology of the sample under an oscillating frequency at 100 Hz resulted from the fluctuation of the grain growth rates (R) during solidification. In beam oscillation welding, the local solid–liquid interface movement, which determines the microstructure at the fusion zone, should be distinguished from the welding speed [29]. The shape of the molten pool is strongly influenced by the laser beam path. Both the laser beam path with the located keyhole and the outline of the molten pool were illustrated in Figure 11. The time values in the images of the molten pool in Figure 9a were chronologically labeled as t1 to t6, respectively. Furthermore, *L* represents the displacement of the solid–liquid interface after 0.004 s. The local grain growth rate (Rdt) is defined as the displacement of interface (L) at the tail of the weld pool per unit time. The path was asymmetrical and involved an overlap of the translation motion of the scanner and the beam scanning motion. From t1 to t3, the solid–liquid interface moved slowly along the welding direction. This could lead to a narrow equiaxed or columnar dendritic microstructure. However, from t4 to t6, the local grain growth rate at the center of the fusion zone rapidly increased, resulting in the dramatic reduction of the molten pool. The path of the beam periodically overlapped, hence the equiaxed region was periodically distributed.

**Figure 12:**
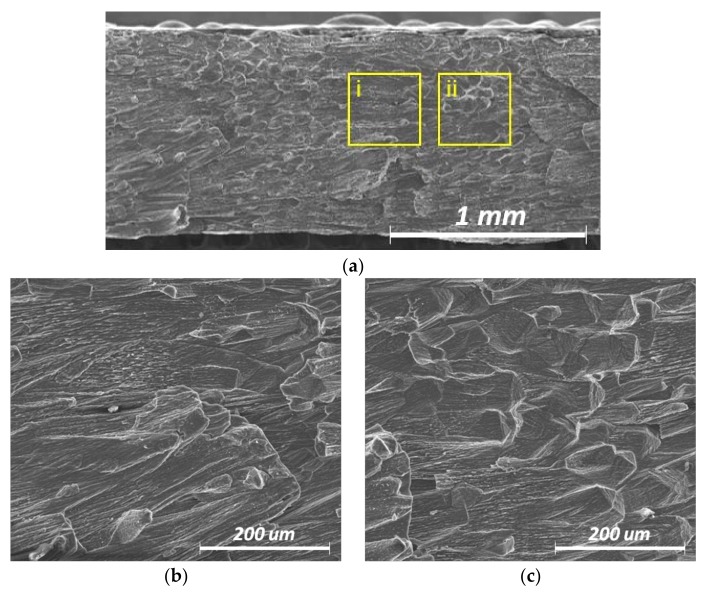
Field-emission scanning electron (FESEM) images of the specimens treated with oscillating laser beams at 100 Hz with the infinite-shaped pattern. (**a**) Fracture surface 1 mm from the start point; (**b**) high-magnification image of the yellow box region (i) in Figure 12a–c high-magnification image of the yellow box region (ii) in Figure 12a.

All the self-restraint test specimens exhibited dendritic and eutectic structures on the fracture surface. Smooth crack surfaces were evident, indicating that a thin liquid layer was present during hot cracking. On the fracture surface of the specimen at 100 Hz with an infinite-shaped beam pattern, the equiaxed and columnar regions alternated periodically (Figure 12). Consequently, the solidification crack susceptibility in oscillating laser beam welding was strongly influenced by the distribution of the equiaxed zone along the centerline. Furthermore, a nonuniform equiaxed zone was preferred, whereas a wide and linear equiaxed zone should be avoided, in accordance with a previous study using low-frequency beam oscillation [31].

## 4. Conclusions

Hot cracking susceptibility during laser welding with the beam oscillation of the automotive aluminum alloy, Al 6014, was evaluated using a self-restraint test. High-speed beam scanning and high laser beam density were implemented by galvanometers and single mode laser, respectively. In most cases, the keyhole moves with the molten pool at a very high speed and the boundary of the pool quasi-steadily transformed with the welding speed. Compared with linear welding, laser welding with beam oscillation resulted in a larger molten pool, and therefore increased cracking susceptibility, because the temperature gradient in the solidification region decreased, widening the equiaxed zone at the center of the fusion zone. However, a longitudinally oscillating bead shape was observed for an infinite-shaped scanning pattern at 100 Hz and 3.5 m/min welding speed. This originated from the sinuous movement of the laser beam along the longitudinal direction. The width of the equiaxed zone at the center of the fusion zone was also fluctuating. Consequently, crack propagation was hindered by this solidification morphology.

## Figures and Tables

**Table 1:** Chemical composition of base material (wt. %).

Chemical Element	Composition (wt. %)
Si	0.09
Fe	0.16
Cu	1.42
Mn	0.04
Mg	2.45
Cr	0.21
Zn	5.61
Ti	0.03
Al	Bal.

**Table 2:** Laser welding conditions for the experiment.

Welding Parameter	Experiment for Circular Pattern	Experiment for Infinite-Shaped Pattern	
Laser power (W)	900		
Welding speed (m/min)	3	3	3.5
Oscillation width (mm)	0, 0.8, 1.6	0, 0.8, 1.6	0, 0.4, 0.8
Oscillation frequency (Hz)	0, 100, 200, 300	200	0, 100, 200, 300
Focal position (mm)	0		
Beam tilting angle (°)	0		
Beam pattern	Linear, Circle	Linear, infinite-shaped	
Shielding gas	Non-shielding		

**Table 3:** Equipment and its specification applied in experiment.

Equipment	Specification
Yb:YAG laser (YLR-1000-SM-WC)	Type: Single-mode laser
	Maximum power: 1 kW
	Wavelength: 1060 nm
	Fiber diameter: 13 μm
Laser optic (D50)	Focal length: 250 mm
	Maximum wobble frequency: 300 Hz
High speed camera (UX50)	Maximum frame rate: 5000 fps
Illumination laser (LIMO120-F400)	Maximum power: 120 W
	Wavelength: 808 nm
